# Slug and E-Cadherin: Stealth Accomplices?

**DOI:** 10.3389/fmolb.2020.00138

**Published:** 2020-07-14

**Authors:** Esta Sterneck, Dipak K. Poria, Kuppusamy Balamurugan

**Affiliations:** Laboratory of Cell and Developmental Signaling, Center for Cancer Research, National Cancer Institute, Frederick, MD, United States

**Keywords:** mammary gland, breast cancer, epithelial-mesenchymal transition (EMT), E-cadherin (CDH1), Slug (SNAI2), basal, luminal

## Abstract

During physiological epithelial-mesenchymal transition (EMT), which is important for embryogenesis and wound healing, epithelial cells activate a program to remodel their structure and achieve a mesenchymal fate. In cancer cells, EMT confers increased invasiveness and tumor-initiating capacity, which contribute to metastasis and resistance to therapeutics. However, cellular plasticity that navigates between epithelial and mesenchymal states and maintenance of a hybrid or partial E/M phenotype appears to be even more important for cancer progression. Besides other core EMT transcription factors, the well-characterized Snail-family proteins Snail (*SNAI1*) and Slug (*SNAI2*) play important roles in both physiological and pathological EMT. Often mentioned in unison, they do, however, differ in their functions in many scenarios. Indeed, Slug expression does not always correlate with complete EMT or loss of E-cadherin (*CDH1*). For example, Slug plays important roles in mammary epithelial cell progenitor cell lineage commitment and differentiation, DNA damage responses, hematopoietic stem cell self-renewal, and in pathologies such as pulmonary fibrosis and atherosclerosis. In this Perspective, we highlight Slug functions in mammary epithelial cells and breast cancer as a “non-EMT factor” in basal epithelial cells and stem cells with focus reports that demonstrate co-expression of Slug and E-cadherin. We speculate that Slug and E-cadherin may cooperate in normal mammary gland and breast cancer/stem cells and advocate for functional assessment of such Slug^+^/E-cadherin^low/+^ (SNAI2^+^/CDH1^low/+^) “basal-like epithelial” cells. Thus, Slug may be regarded as less of an EMT factor than driver of the basal epithelial cell phenotype.

## Introduction

Phenotypic plasticity refers to the ability of cells to change their phenotype such as transitioning from epithelial to mesenchymal characteristics or from stem cell to a differentiated state. This plasticity may be one-directional or reversible and transient or permanent. In addition, cells may inhabit any state between such defined phenotypes in a stable or metastable manner. The cellular plasticity of cancer cells relies on molecular mechanisms from the playbook of normal embryonic or postnatal development. The mammary gland is a particularly dynamic organ undergoing expansion and differentiation during pregnancy and early lactation, followed by cell death and remodeling during the course of weaning (Richert et al., 2000; Shamir and Ewald, 2015). One type of plasticity, the epithelial-mesenchymal transition (EMT) is an important process for normal development and tumor biology. During EMT cells lose their epithelial polarization and organization and E-cadherin expression is drastically reduced through active inhibition of gene expression (Micalizzi et al., 2010). Thus, E-cadherin downregulation is often used as a (surrogate) marker for EMT. Snail and Slug are two transcription factors that can directly repress the E-cadherin gene (*CDH1*) promoter while activating the promoters of key mesenchymal genes such as ZEB1 and vimentin (Ye et al., 2015; Xu et al., 2019). For comprehensive background information, we refer the reader to a number of excellent recent reviews, which summarize Slug functions and regulation of expression (Zhou et al., 2019), regulation by posttranslational modifications (Xu et al., 2019), and the non-redundant functions of EMT factors (Stemmler et al., 2019).

Snail and Slug are often named in unison as if functionally synonymous, and expression of Slug alone suggested as indication of a mesenchymal gene program. However, the endogenous functions of Snail and Slug can vary significantly, in part due to differences in DNA-binding affinity and interaction partners. Thus, Slug and Snail have overlapping (e.g., *CDH1, VIM*) as well-distinct sets of target genes (e.g., *L1CAM, PTEN*) (Stemmler et al., 2019; Xu et al., 2019). Slug plays a role in maintaining the structure of the normal mammary gland and modulates the specific phenotypes of breast cancer subtypes (Phillips and Kuperwasser, 2014). Overexpression of ectopic Slug may lead to cellular responses that mimic Snail functions, such as inhibition of *CDH1* gene expression. However, at physiological levels, Slug and E-cadherin are often co-expressed. Thus, results from overexpression studies and cell culture paradigms, as has been noted before (Alves et al., 2009), have created the perception of Slug as an EMT transcription factor, when many times it is not. The above-mentioned reviews provide numerous examples for the role of Slug in EMT. Whether Slug can execute this role in the absence of its partner Snail, has perhaps not been addressed in detail. In experimental systems where Slug “inhibits expression of E-cadherin,” it may be reduced but not abolished (e.g., Leong et al., 2007). The co-occurrence of Slug and E-cadherin may be particularly relevant for hybrid EMT and cellular plasticity, which are being recognized as important factors in cancer progression (Jolly et al., 2018; Aiello and Kang, 2019; Gupta et al., 2019), along with the role of E-cadherin in not only the establishment of metastases but also the process of dissemination (Rodriguez et al., 2012; Padmanaban et al., 2019; Voglstaetter et al., 2019). In this Perspective, we want to highlight examples of co-expression of Slug and E-cadherin and hypothesize on its relevance for tumor biology.

## Slug Promotes the Basal Cell Phenotype and Stemness in the Mammary Epithelium: Not Without E-Cadherin?

The mammary gland epithelium is a bilayer of luminal epithelial cells and basal/myoepithelial cells that express unique sets of cytokeratins. Within each layer are subsets of cells with different characteristics based on e.g., expression of specific steroid hormone receptors and stem cell or lineage progenitors properties (Visvader and Stingl, 2014). To our knowledge, Slug protein expression has not been investigated in normal human mammary stem/progenitor cells. Mouse models have, however, provided significant insights about Slug's function in development. Slug is expressed in basal mammary epithelial cells (MECs) and is the only EMT factor that is enriched in both mouse and (by mRNA) human mammary stem cells (MaSC) that reside within this compartment (Lim et al., 2010; Guo et al., 2012; Nassour et al., 2012). Interestingly, *SNAI2*/Slug mRNA expression is detectable in human luminal progenitors (albeit at significantly lower levels compared to basal cells) but not in their mouse counterpart (Lim et al., 2010). Its functional significance has yet to be determined but may be relevant for the development of luminal breast cancer (see below). Slug plays an important role in maintaining stemness in cooperation with proteins such as Sox9 and the chromatin modifier LSD1 (Guo et al., 2012; Phillips et al., 2014; Bai et al., 2017). In addition, Slug determines progenitor cell lineage commitment and differentiation by actively repressing the luminal cell state (Phillips and Kuperwasser, 2014). Snail, on the other hand, is expressed in the mesenchymal stromal fibroblasts surrounding the mammary duct and not in normal mammary epithelial cells (Nassour et al., 2012; Ye et al., 2015). P-cadherin (*CDH3*), the classical myoepithelial cadherin (Shamir and Ewald, 2015), is a target gene of Slug and mediates many of its functions (Idoux-Gillet et al., 2018). E-cadherin is highly expressed in luminal cells, but Slug expressing basal cells also express E-cadherin (Ye et al., 2015). E-cadherin localizes to the lateral cell-junctions. Basal cells and luminal cells are very different in size and shape. Most likely, normal cells engage feedback mechanisms to regulate the levels of E-cadherin based on their cell-cell contacts. How should one compare the “functionally equivalent” amounts of E-cadherin cell-cell adhesions? For these reasons, here we use the term “E-cadherin+” to refer to cells that express any detectable amount of the protein.

**Figure 1:**
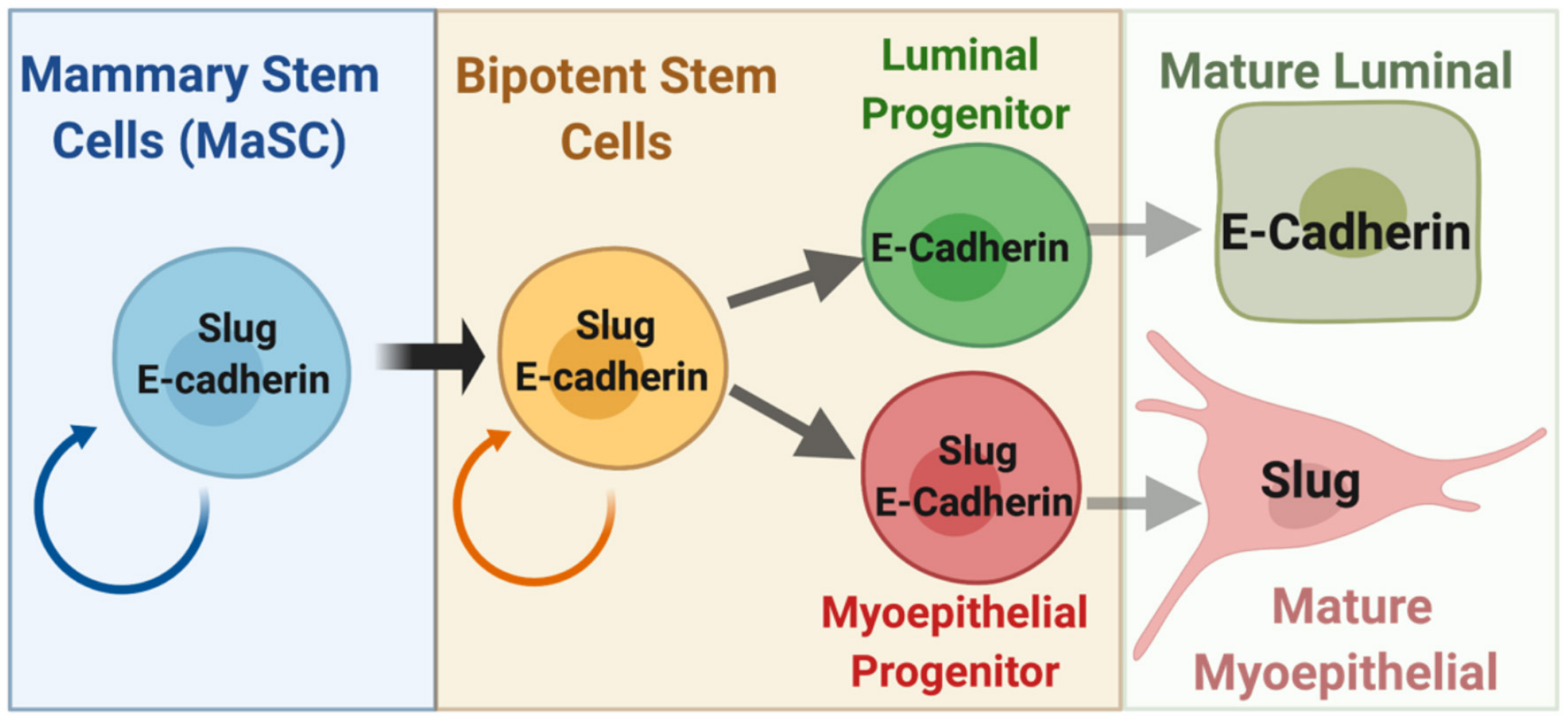
Schematic of the mammary epithelial stem cell hierarchy depicting the known and proposed relationships of Slug and E-cadherin (see text for details). Relative differences in expression levels between cells can be assumed but are not depicted. Figure was created with BioRender.com.

Surprisingly, Slug-deficiency does not impair the regeneration capacity of transplanted mammary tissue fragments although lineage dynamics were compromised (Nassour et al., 2012). However, when the tissue was dissociated, the organoid-forming and gland-reconstituting activities of stem cells are dependent on Slug (Guo et al., 2012; Phillips et al., 2014). The apparent paradox might be explained by a pro-survival function of Slug in stem cells that becomes apparent in the dissociation paradigm and could also be relevant for cancer stem cell assays. Whether E-cadherin plays a role in MaSCs is not known (Figure 1). However, E-cadherin is important for pluripotency in embryonic stem cells through cooperation with the Wnt signaling pathway (Pieters and van Roy, 2014). Studies of the mechanisms leading to expansion of the mammary gland during pregnancy revealed that a TGFβ2/integrin-αvβ3 pathway induces Slug protein accumulation in MaSCs without affecting mRNA expression or overt EMT signatures. Knockdown of αvβ3 in MDA-MB-231 cells reduced Slug expression and compromised survival of tumor initiating cells (Desgrosellier et al., 2014). In addition, Slug has a role in genome maintenance. *Slug* knockout mice exhibited premature aging of mammary epithelium with loss of mammary stem cell activity, luminal differentiation of basal cells, and increased DNA damage due to replicative stress (Gross et al., 2019). Conceivably, this function could also contribute to cancer stem cell maintenance and resistance to chemotherapeutics. Unexpectedly though, Slug knockout impairs MEC death during post-lactational mammary gland involution (Castillo-Lluva et al., 2015). The contrast of functions in developmental cell death vs. promoting cancer cell survival is not unique to Slug but also seen with STAT3 and C/EBPδ transcription factors (Balamurugan and Sterneck, 2013; Resemann et al., 2014). In summary, studies in mouse models demonstrate that Slug determines a basal MEC phenotype and promotes mammary stem cell self-renewal, genomic maintenance and cell survival, all of which is at least compatible with E-cadherin expression.

## Slug and Breast Cancer Stem Cells: Which Ones, and What About E-Cadherin?

Breast cancer (BC) is classified into subtypes based on expression of hormone receptors and HER2, which are usually associated with a luminal cell phenotype. Triple negative breast cancer (TNBC) lacking expression of these markers presents mostly with a basal or basal-like BC (BLBC) phenotype. Mesenchymal markers are enriched in a subset of TNBCs and are correlated with stemness properties (Dai et al., 2016). Despite controversies surrounding the cancer stem cell (CSC) theory, the concept has contributed to the identification of cancer cell plasticity and important mechanisms underlying tumor progression (Wang et al., 2015). Various cell surface molecules (e.g., CD44, CD24, CD133) and combinations thereof as well as ALDH activity have been used to enrich for cells with stemness properties and their frequency varies by BC subtype (Rodriguez et al., 2019). The CD44^+^/CD24^−/low^ CSCs are mesenchymal-like while ALDH1^+^ and CD44^+^/CD24^+^ stem cells are epithelial-like. In node-positive BC, co-occurrence of ALDH1 and Slug in primary lesions was associated with shorter disease-free survival, though co-expression at the single cell level was not assessed (Ito et al., 2016). Transcriptomic analysis of patient-derived xenograft models showed that *SNAI2*/Slug mRNA was enriched in the mesenchymal CSCs “consistent” with its classification as an “EMT factor” (Liu et al., 2018). However, low levels of mRNA do not preclude Slug protein expression as shown for HMLER hybrid E/M cells (Kroger et al., 2019). Slug expression and its role in distinct types of tumor initiating cells with low proteasome activity, high STAT3, or SOX2/OCT4 activity has not been investigated (Vlashi et al., 2013; Wei et al., 2014; Tang et al., 2015). However, Slug was shown to be important for survival of integrin αvβ3/Src-induced CSCs that also express E-cadherin and exist across BC subtypes (Sun et al., 2018). Mesenchymal CD44^+^/CD24^−/low^ CSCs do not express E-cadherin but gain further tumor initiating capacity with the expression of the epithelial adhesion molecule EpCAM that marks “hybrid E/M” states (Dittmer, 2018). In BLBC cell lines, the p63 transcription factor, which is important for MaSCs (Memmi et al., 2015), promotes invasiveness through Slug without compromising E-cadherin expression (Dang et al., 2015). It is thus conceivable that E-cadherin may be important for a subset of breast CSCs. E-cadherin promotes BC cell mammosphere formation, a measure of stem cell self-renewal (Manuel Iglesias et al., 2013). E-cadherin can promote stemness in lung and gastric cancer cells (Tang et al., 2019; Ye et al., 2020) and signaling pathways that are known to support CSCs such as by EGFR (Rodriguez et al., 2012; Steelman et al., 2016), LIFR (del Valle et al., 2013), and Wnt (Pieters and van Roy, 2014). To our knowledge, the expression and potential function of E-cadherin in different types of BC stem cells has not been analyzed to date.

## Brief Update on Slug in Breast Cancer: Quite Basal and to the Bone—Along With E-Cadherin?

Not surprisingly, Slug expression is preferentially observed in basal/TNBC as are mesenchymal and stemness markers. Compelling evidences for an important role of Slug in human breast cancer and mechanistic underpinnings have been reviewed (Phillips and Kuperwasser, 2014; Zhou et al., 2019). Here, we want to point out that the majority of basal/TNBC cancers do, however, not lose E-cadherin expression (Rodriguez et al., 2012; Horne et al., 2018). In support of the dissociation of Slug from the EMT processes, expression of Slug protein or E-cadherin (*CDH1*) mRNA were not correlated with the activation of a core EMT gene expression signature in breast cancer (Savci-Heijink et al., 2019). However, aberrant expression of Slug explains the emergence of basal tumor phenotypes from luminal progenitors (Phillips and Kuperwasser, 2014), or conversion of a luminal to basal phenotype through TGFβ (Sflomos et al., 2016). Furthermore, Slug contributes to treatment resistance of luminal cancers in part through promoting a phenotypic shift to a basal phenotype such as in HER2+ cells (Oliveras-Ferraros et al., 2012) and ER+ cells (Tsou et al., 2015; Geng et al., 2016; Alves et al., 2018). In addition, Slug expression in ER+ BC cell lines also promotes mammosphere formation, proliferation and invasive properties (Storci et al., 2010; Chimge et al., 2011; Mendoza-Villanueva et al., 2016; Manne et al., 2017). Interestingly, although *CDH1* mRNA levels increased with Slug knockdown in drug-resistant MCF-7 cells, total E-cadherin protein levels did not (Alves et al., 2018). A negative feedback loop between Slug and ER is seen in ER+ breast cancer cell lines, where estrogen inhibits TGFβ-induced EMT by suppressing Slug but not Snail expression (Liu et al., 2019). In the context of RUNX2/TGFβ/Wnt-signaling, a balanced expression of Slug and ERα is implicated in bone metastasis of ER+ BC cell lines (Chimge et al., 2011). Furthermore, in TNBC cell lines, Slug promotes bone metastasis but not lung infiltration (Ferrari-Amorotti et al., 2014). Given the implications of integrin αvβ3 in bone metastasis of various epithelial cancers (Kwakwa and Sterling, 2017), above-mentioned role of Slug in the integrin αvβ3^+^ breast CSCs that do express E-cadherin (Sun et al., 2018), and elevated E-cadherin expression in BC bone metastases (Saha et al., 2007; Matteucci et al., 2013), we hypothesize that E-cadherin expressing αvβ3^+^/Slug^+^ stem-like cells could play a significant role in breast cancer bone metastasis.

## Slug and EMT: Guilty by Association?

Without doubt, Slug's cousin Snail is a potent mediator of EMT. Slug and Snail are often coordinately expressed (Katoh, 2011), and Slug can thereby be implicated in EMT as “caught at the scene.” For example, in breast cancers that show correlation of Slug and Snail with lymph node metastasis, only Slug expression was seen in more histologically semi-differentiated structures. The observation led the authors to the hypothesis (foresight?) that each drives distinct tumor invasion modes (Come et al., 2006). Investigations of the mouse MMTV-PyMT tumor model showed that Snail expressing cells are mesenchymal while Slug expressing cells exhibited an epithelial phenotype. Despite a large number of common target genes, only Snail occupied the promoters of key mesenchymal marker genes (Ye et al., 2015). In MDA-MB-231 cells, Snail was necessary for binding of Slug to the ZEB1 promoter and its activation indicating that Slug alone may not drive EMT in the absence of Snail (Ye et al., 2015). On the other hand, Slug can *attenuate* E-cadherin levels indirectly by post-transcriptional mechanisms through miR-221 and by promoting protein degradation (Pan et al., 2016; Anzai et al., 2017). Using oncogene-transformed human mammary epithelial cells (HMLER), Kroger et al. showed that Slug protein expression in such epithelial cells was similar to that in mesenchymal and hybrid E/M cells. Only epithelial cells expressed E-cadherin. Mesenchymal cells had the highest levels of ZEB1, while hybrid E/M cells exhibited the most Snail expression along with CSC activity. Interestingly, hybrid E/M cells showed significant downregulation of Slug mRNA but no change at the protein level, suggesting significant stabilization of Slug protein in these cells (Kroger et al., 2019). While the mRNA data are consistent with reports that Snail can repress Slug/*SNAI2* expression (Sundararajan et al., 2019), such results illustrate the importance of protein data even when mRNA expression is downregulated. Indeed, several mechanisms for stabilization of the Slug protein have been reported (Xu et al., 2019; Zhou et al., 2019). In the MMTV-PyMT mouse tumor model, Slug^+^ populations also express E-cadherin and a subpopulation of Slug^+^ cells also express EpCAM. Immunocytochemistry showed at the single cell level that among human basal breast cancer cell lines, there are various percentages of single and double positive cells for Slug and Snail protein (Ye et al., 2015). E-cadherin was not evaluated here, but these may be good models to mechanistically dissect the expression and function of E-cadherin in Slug^+^ cells.

The specific position of a cell along the E-M continuum may depend in part on the expression levels of Snail vs. Slug and their fine-tuning of E-cadherin expression levels. Mutual regulation of Snail and Slug has also been described in other cell types. Snail inhibits Slug in ovarian cancer cell lines, i.e., Slug is downregulated during EMT (Sundararajan et al., 2019). Snail and Slug engage in mutual negative feedback of expression during bone development (Chen and Gridley, 2013). In oral squamous carcinoma cell lines, Snail and Slug can engage in mutual attenuation of expression although both are induced by TGFβ (Nakamura et al., 2018). Lastly, Slug can support its own gene transcription in cooperation with Sox9 during embryonic development, i.e., when SOX9 is induced by BMP and Wnt signaling, Slug expression self-amplifies (Sakai et al., 2006). These types of feedback regulation may not only balance their relative expression levels but play a role in generating a metastable cell phenotype with Slug/Snail ratios performing the function of an E/M rheostat and tuning the expression level of E-cadherin.

## Discussion: What are the frontiers?

A comprehensive analysis of Slug's prognostic/predictive biomarker potential and correlation with E-cadherin expression at single cell resolution with well-validated antibodies is still outstanding. Stratification by subtype and additional clinical criteria and biomarkers will be essential to gain significant insight. Because nuclear expression of Slug has also been correlated with cytoplasmic E-cadherin staining (Prasad et al., 2009), subcellular resolution may be important as well as consideration of E-cadherin isoforms (Ye et al., 2013; Konze et al., 2014; Wu et al., 2016). Similarly, sensitive single cell resolution analysis of Slug and E-cadherin *protein* expression among the diversity of cells in the mouse and human mammary epithelium and breast cancer may bring about new frontiers for functional studies.

**Figure 2:**
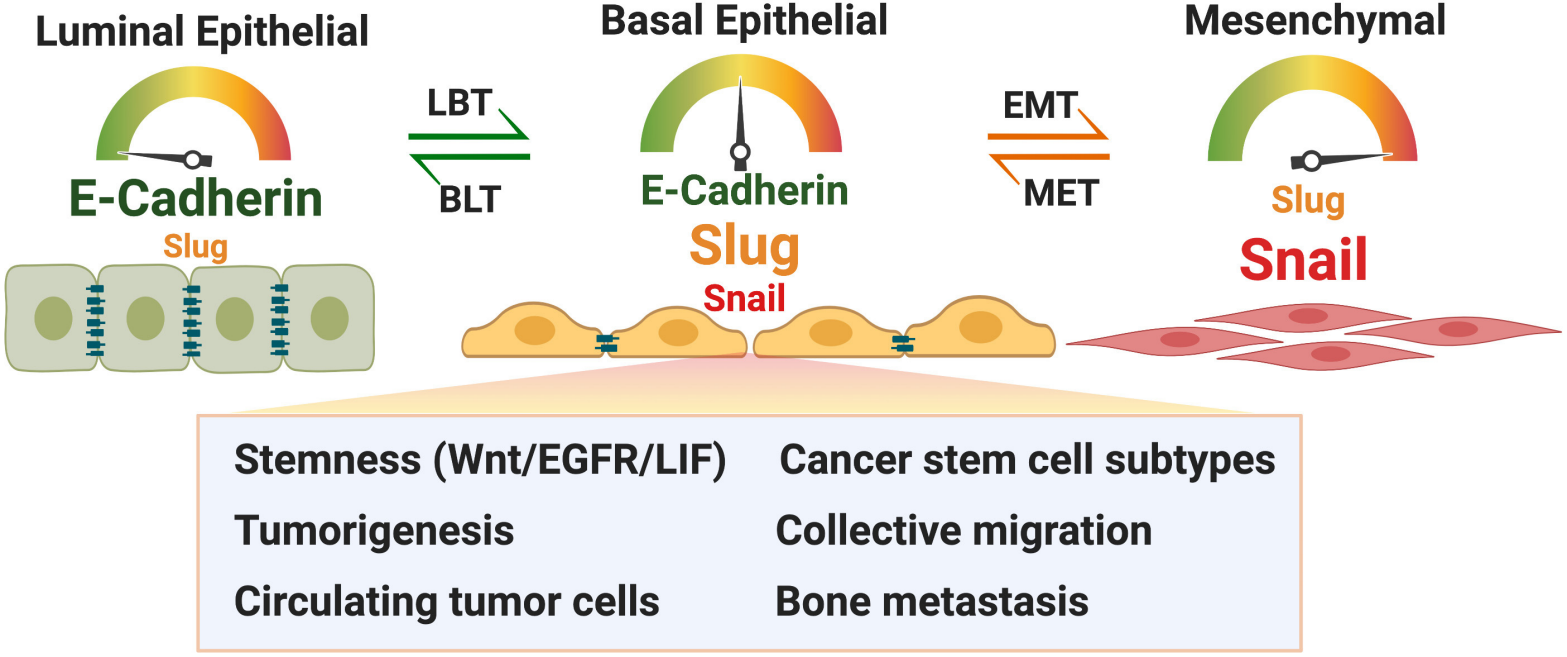
Model describing expression of E-cadherin and Slug in luminal epithelial, basal epithelial and mesenchymal cancer cells and the proposed qualities of basal epithelial cancer cells due to co-expression of Slug and E-cadherin. Luminal basal transition (LBT) and basal luminal transition (BLT) are proposed terminologies in addition to EMT and MET. See text for details. Figure was created with BioRender.com.

Figure 2 summarizes cancer cell-related hypotheses on E-cadherin expression in relation to Slug and their potentially cooperative contribution to cancer progression. Due to the limited scope of this Perspective, the many other factors that are known to modulate these phenotypes were not included. A cell that expresses a moderate level of E-cadherin and Slug may be in a particular goldilocks state that facilitates these functions. Increasingly, a role for E-cadherin in cancer cell dissemination is being recognized (see Introduction). Collective migration/dissemination is one aspect in which Slug and E-cadherin may cooperate (Dang et al., 2015), and Slug^+^/E-cadherin^+^ cells may be particularly relevant in metastasis to the bone. In these contexts, the E-cadherin^+^ cell may not be expressing high but still functionally relevant levels of E-cadherin. As hybrid E/M phenotypes in circulating tumor cells (CTCs) reveal strong association with tumor-initiation potential and metastasis (Fabisiewicz et al., 2020), Slug^+^/E-cadherin^+^ cells are likely contributors to disseminating CTCs as well, perhaps in part through inhibition of anoikis or E-cadherin's potential to support stemness promoting signaling pathways.

The epithelial cadherin EpCAM has received much attention for its expression and functions in tumor cells (Dittmer, 2018). It is time that E-cadherin emerges from its shadow and sheds the prevailing image of being (only) a tumor suppressor. Considering mesenchymal vs. epithelial state and luminal vs. basal state along with time of development (of the organ or tumor) and space (microenvironment), cells navigate at least these six dimensions to attain a particular phenotype, challenging our need for classification. Regard for Slug^+^/E-cadherin^+/low^ cells may in part address this challenge and contribute to better understanding of cancer biology.

## Author Contributions

ES and KB performed literature research and wrote the manuscript. DP reviewed literature, co-wrote the manuscript, and generated the figures. All authors contributed to the article and approved the submitted version.

## Conflict of Interest

The authors declare that the research was conducted in the absence of any commercial or financial relationships that could be construed as a potential conflict of interest.
